# Case Report: A case of TAFRO syndrome with severe and prolonged thrombocytopenia: diagnostic pitfalls

**DOI:** 10.3389/fimmu.2023.1266187

**Published:** 2023-10-13

**Authors:** Hironori Sato, Atsuhiro Kanno, Minato Sato, Akari Endo, Hiroki Ito, Takahiro Ohara, Yuko Shirota, Kazuhiro Sumitomo, Takefumi Mori, Katsutoshi Furukawa

**Affiliations:** ^1^ Division of Geriatric and Community Medicine, Faculty of Medicine, Tohoku Medical and Pharmaceutical University, Sendai, Japan; ^2^ Division of Nephrology and Endocrinology, Faculty of Medicine, Tohoku Medical and Pharmaceutical University, Sendai, Japan; ^3^ Division of Hematology and Rheumatology, Faculty of Medicine, Tohoku Medical and Pharmaceutical University, Sendai, Japan; ^4^ Department of Community and General Medicine, Tohoku Medical and Pharmaceutical University, Wakabayashi Hospital, Sendai, Japan

**Keywords:** acute kidney injury, TAFRO syndrome, disseminated intravascular coagulation, immune thrombocytopenia, thrombotic microangiopathy

## Abstract

Thrombocytopenia, anasarca, myelofibrosis, renal dysfunction, and organomegaly (TAFRO) syndrome is a rare condition with diverse clinical and pathological characteristics related to multi-organ damage. We report a case of TAFRO syndrome complicated by immune thrombocytopenia with prolonged fever and thrombocytopenia for several weeks. A 61-year-old man was transferred with sepsis caused by Enterococcus faecalis, and developed disseminated intravascular coagulation. Antibiotics treatment was initiated: however, low-grade fever and thrombocytopenia persisted despite the adequate antimicrobial treatment. Systemic edema, pleural effusion, and ascites had developed before hospitalization, and renal and liver function had deteriorated, resulting in progressive multi-organ damage. Prednisolone 40 mg/day was initiated based on the assumption of a condition in which excessive production of inflammatory cytokines would lead to systemic deterioration and fatal organ damage. Subsequently, the fever resolved, and renal function began to normalize. However, thrombocytopenia did not show much recovery trend after Helicobacter pylori eradication therapy and initiation of thrombopoietin receptor agonists. Bone marrow biopsy results showed normal bone marrow with no malignant findings. Alternatively, significant clinical signs met the diagnostic criteria for TAFRO syndrome, and a renal biopsy revealed thrombotic microangiopathy, which is also reasonable for renal involvement in TAFRO syndrome. The use of cyclosporine remarkably corrected the thrombocytopenia. We considered this a case of TAFRO syndrome that developed after sepsis with disseminated intravascular coagulation and performed the differential diagnosis of prolonged thrombocytopenia and excluded it. Although TAFRO syndrome is a unique disease concept, diagnostic criteria may consist of nonspecific elements such as generalized edema, thrombocytopenia, persistent fever, and elevated inflammatory response, and there are many differential conditions to exclude, requiring caution in diagnosing TAFRO syndrome.

## Introduction

Thrombocytopenia, anasarca, myelofibrosis, renal dysfunction, and organomegaly (TAFRO) syndrome was first reported in 2010 (1). The clinical and pathological characteristics thereof are diverse and related to multi-organ damages, and the diagnostic criteria were updated in 2019 (2). TAFRO syndrome is a clinical entity established after excluding diseases, such as hematologic malignancies, autoimmune diseases, and infections. In addition to the fact that the pathogenesis and pathophysiology of TAFRO syndrome are not still elucidated, non-specific factors such as fluid retention, thrombocytopenia, fever, and high inflammatory response are included in diagnostic criteria, which are also seen in severe infections and malignancies. These make the diagnosis even more complicated, and differential diagnosis is also complex. In the present case, it was required particular care in differentiating disseminated intravascular coagulation syndrome (DIC) from severe infections or autoimmune disease such as systemic lupus erythematosus (SLE) (3). We specifically excluded persistent bacteremia with frequent blood cultures and we tried to diagnose TAFRO syndrome in a situation that eliminated the influence of DIC associated with sepsis, which was present at admission. We report a case of DIC complicated with intestinal infection, followed by prolonged and severe thrombocytopenia despite adequate antimicrobial therapy and sustained systemic edema. It was difficult to rule out various differential diagnoses, such as DIC associated with infection itself or SLE as a differential for fever and thrombocytopenia; nevertheless, the diagnosis of TAFRO syndrome was ultimately made.

## Case presentation

**Figure 1:**
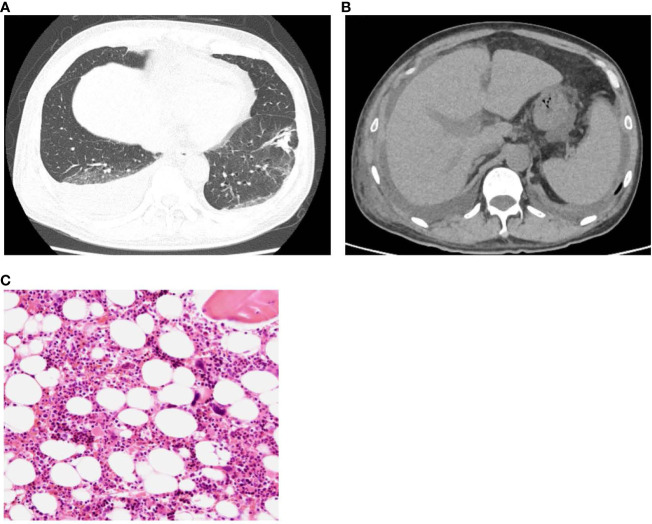
**(A)** Plain computed tomography revealed a post-pneumonia change in the lower lobe of the left lung in the process of healing, and **(B)** right-dominant bilateral pleural effusions, hepatosplenomegaly. **(C)** Normocellular marrow with mature megakaryocytes, but no fibrosis. (H&E staining, x100).

A 61-year-old man was admitted to a local hospital with acute kidney injury (AKI) and thrombocytopenia after recovering from Legionella pneumonia. He also had a low-grade fever with elevated inflammatory blood markers despite antibiotics treatment, which was discontinued because of lack of clinical efficacy. He was transferred to our hospital for further examination and treatment for recurrent AKI, watery diarrhea, and fever of unknown origin (FUO). Upon admission, his body temperature was 37.6°C, and other vital signs showed no abnormalities. Physical examination revealed conjunctival anemia, abdominal distention, and tenderness in the midline; however, rebound tenderness was not observed. Systemic edema was noted during admission to the previous hospital, and diuretics were used. At admission to our hospital, the edema in the extremities and trunk remained residual. Laboratory findings on admission (**Table 1**) were as follows: hemoglobin of 8.7 g/dL; platelet count, 8,000/μL; serum creatinine, 3.26 mg/dL; estimated glomerular filtration rate (eGFR), 16.4 mL/min/1.73m^2^; alkaline phosphatase, 427 U/L; C-reactive protein, 14.56 mg/dL; and procalcitonin,4.22 ng/mL. Both antineutrophil cytoplasmic antibody and rheumatoid factor tests were negative. Serum levels of interleukin-6 (IL-6) and the plasma levels of vascular endothelial growth factor (VEGF) were 21.9 pg/mL (reference range, < 4.0) and 426 pg/mL (reference range, 143.1 - 658.8), respectively. Direct and indirect Coombs tests results were negative. Haptoglobin levels did not decrease, and schistocytes were not detected in the peripheral blood smear. Because the activity of a disintegrin-like and metalloproteinase with thrombospondin type 1 motifs 13 (ADAMTS13) was maintained at 53%, the possibility of thrombotic thrombocytopenic purpura was unlikely. Coagulation analysis showed abnormalities including increased levels of fibrin/fibrinogen degradation products (FDP) of 100.3 µg/mL, D-dimer of 44.74 µg/mL, and thrombin-antithrombin complex (TAT) of 9.5 µg/mL. These laboratory findings met the diagnostic criteria for DIC (4). Additionally, an increase of 5 points in the Sequential Organ Failure Assessment score strongly suggested sepsis based on its definition (5). Urinary findings were a trace proteinuria and mild microhematuria. Computed tomography revealed a post-pneumonia change in the lower lobe of the left lung during healing, right-dominant bilateral pleural effusions (**Figure 1A**), hepatosplenomegaly (**Figure 1B**), and ascites. Bone-marrow aspiration performed at a previous hospital revealed no phagocytic cells, indicating a low possibility of hemophagocytic syndrome.

**Table 1:** Laboratory findings at transfer to our hospital.

Complete Blood Count		Blood Chemistry		Immunology	
White blood cell	7.7x10^3^/µL	Total protein	4.7 g/dL	IgG	1050 mg/dL
Neutrophil	87.0%	Albumin	1.4 g/dL	IL-6	21.9 pg/mL
Lymphocyte	2.0%	Total bilirubin	0.96 mg/dL	C3	102 mg/dL
Monocyte	8.0%	Direct bilirubin	0.61 mg/dL	C4	25 mg/dL
Eosinophil	0.0%	Aspartate aminotransferase	20 U/L	Rheumatoid factor	10 IU/mL
Basophil	2.0%	Alanine aminotransferase	4 U/L	Anti-nuclear antigen	<40
Red blood cell	2.93×10^6^/µL	Lactate dehydrogenase	238 U/L	Anti-double strand-DNA	<12 IU/mL
Hemoglobin	8.7 g/dL	Alkaline phosphatase	427 U/L	MPO-ANCA	<1.0 U/mL
Hematocrit	26.4%	γ-Glutamyl transpeptidase	104 U/L	PR3-ANCA	<1.0 U/mL
Mean corpuscular volume	90.4 fL	Creatine phosphokinase	35 U/L	Anti-H.pylori-antibody	20 U/mL
Reticulocyte	2.7%	Blood urea nitrogen	60 mg/dL	Platelet associated-IgG	613 IU/mL
Platelet	8.0×10^3^ / µL	Creatinine	13.26 mg/dL	FLC ratio (kappa/lambda)	1.78
ESR	90 mm/h	eGFR	16.4 mL/min/1.73m^2^	ADAMTS13 activity	53%
**Coagulation**		Uric acid	11.9 mg/dL	Serum amyloid A	1080 µg/mL
Prothrombin time	66.6%	Na	134 mmol/L	Haptoglobin	326 mg/dL
PT-INR	1.32	K	4.4 mmol/L	**Urinalysis**	
APTT	45.7 sec	Cl	104 mmol/L	pH	5
FDP	100.3 µg/mL	C-reactive protein	14.56 mg/dL	Special gravity	1.017
D-dimer	44.74 µg/mL	Procalcitonin	4.22 ng/mL	Protein	1+
TAT	9.5 µg/mL	Soluble IL-2R	1610 U/mL	Urinary-White blood cell	1-4/hpf
PIC	5.6 µg/mL	VEGF	426 pg/mL	Urinary-Red blood cell	1-4/hpf
Fibrinogen	495 mg/dL	Ferritin	1705 ng/mL	Cast	granular 2+

ESR, erythrocyte sedimentation rate; PT-INR, prothrombin time-international normalized ratio; APTT, activated partial thromboplastin time; FDP, fibrinogen/fibrin degradation products; TAT, thrombin-antithrombin complex; PIC, plasmin-α2 plasmin inhibitor complex; eGFR, estimated glomerular filtration rate; IL-2R, interleukin-2 receptor; VEGF, vascular endothelial growth factor; MPO-ANCA, myeloperoxidase-anti-neutrophil cytoplasmic antibodies; PR3-ANCA, proteinase-3-anti-neutrophil cytoplasmic antibodies; H.pylori, helicobacter pylori; FLC, free light chain; ADAMTS13, a disintegrin-like and metalloproteinase with thrombospondin type 1 motifs 13.

Blood cultures at admission were positive for Enterococcus faecalis, which was considered to be derived from enteral bacterial translocation as a result of persistent diarrhea. Piperacillin/tazobactam, a broad-range penicillin, was initiated for the treatment of bacteremia, followed by de-escalation to ampicillin based on antimicrobial susceptibility. During this period, recombinant thrombomodulin was initiated in addition to antibiotics for DIC, which was considered incidental to the bacteremia; however, the coagulation abnormalities did not improve during the first eight weeks after hospitalization. The patient also showed persistently elevated inflammatory markers in the blood, progressive thrombocytopenia, and deteriorated renal function, regardless of these treatments for approximately eight weeks. Based on the worsening general conditions and critical organ damages, we assumed a pathological condition such as a life-threatening systemic inflammatory response syndrome inducing elevation of cytokines and immune cell hyperactivation. We initiated oral prednisolone 40 mg/day (0.5 mg/kg/day) to control systemic inflammation in addition to red blood cell and platelet transfusions for anemia and severe thrombocytopenia, respectively. Nevertheless, platelet counts remained between 8,000 and 18,000 despite platelet transfusions and the additional prior treatments.

Fever with elevated inflammatory markers gradually decreased, and renal function slightly recovered after starting corticosteroids; however anemia and thrombocytopenia did not improve. The patient had edema of the extremities and trunk, especially significant pleural effusion, and ascites, regardless of diuretic prescription at his previous hospital. Persistent bacteremia was ruled out by a sufficient period of broad-spectrum antimicrobial therapy and several blood culture tests, but anemia and thrombocytopenia persisted. In addition, the patient’s fever resolved after the start of moderate-dose steroids, and other vital signs were stable, an illness script different from the clinical course of DIC. The presence of severe thrombocytopenia, pleural effusion, and ascites, which persisted after the start of steroids, was suspicious for TAFRO syndrome. Additionally, the patient fulfilled the four major (thrombocytopenia, anasarca, fever, and organomegaly) and one minor (renal insufficiency) diagnostic criteria for TAFRO syndrome (2).

As a differential diagnosis for severe thrombocytopenia, immune thrombocytopenia (ITP) was assumed, therefore, a thrombopoietin receptor agonist was initiated. Furthermore, Helicobacter pylori (H.pylori) eradication therapy was performed after the confirmation of a positive antibody test result. Subsequently, platelets count slightly increased to greater than 30,000/μL, a level not typically seen in ITP patients responding to treatment.Repeated bone marrow biopsy after these treatments showed normal bone marrow hematopoietic cells and mature megakaryocytes with no malignant findings (**Figure 1C**), and the mildly elevated plasma thrombopoietin level was observed.

Before admission to our department, the patient presented with AKI, which was partially explained by the prerenal causes of prolonged diarrhea. The urinary findings were not highly suggestive of active nephritis; however, trace proteinuria and mild microhematuria were gradually improved and his renal function began to recover with corticosteroid administration. We consulted the rheumatologist to rule out autoimmune disease, essential for diagnosing TAFRO syndrome. Most autoimmune diseases can be ruled out by symptoms, clinical course, and disease-specific autoantibodies; however, it was necessary to consider the possibility of SLE as a differential disease for severe thrombocytopenia with fever. The nephrologist requested a renal biopsy to rule out lupus nephritis, as renal injury was also suggested.

**Figure 2:**
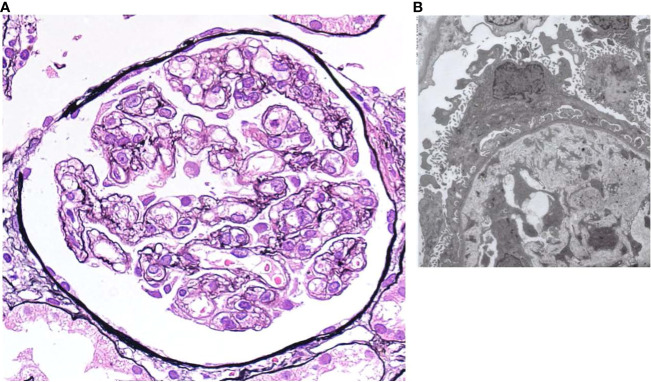
**(A)** Light microscopy shows diffuse global endocapillary proliferative changes with endothelial swelling and double contours in capillary walls (periodic acid–Schiff staining, × 400). **(B)** Electron microscopy shows endothelial cell swelling with expansion of the subendothelial space, and no immune complex deposition is observed in glomeruli.

Therefore, a renal biopsy was planned to clarify the cause of renal injury and to be performed when the platelet count recovered to a level with a reduced risk of complications, including bleeding. The most prominent renal biopsy findings were doubling of the glomerular basement membrane, mesangial and endocapillary proliferation, and subendothelial space expansion in light microscopy images (**Figure 2A**). Interstitial fibrosis, tubular atrophy, and cellular infiltration were observed in some focal areas, and a cellular crescent was formed in one glomerulus. Small arteries or arterioles showed slight endothelial swelling, fibroelastosis, and hyaline arteriolosclerosis. C3 and C1q were positive for capillary loop, whereas IgG, IgM, and kappa and lambda chains were negative for immunofluorescence in the glomerular capillary wall. The involvement of immune complex deposition was considered negative; therefore, we determined that lupus nephritis could be ruled out. Additionally, these findings were not disease-specific but were consistent with renal thrombotic microangiopathy (TMA), a characteristic result of renal involvement in TAFRO syndrome. Electron microscopy showed endothelial cell swelling with expansion of the subendothelial space, and no immune complex deposition was observed in glomeruli (**Figure 2B**). These findings were considered reasonable for TMA.

**Figure 3:**
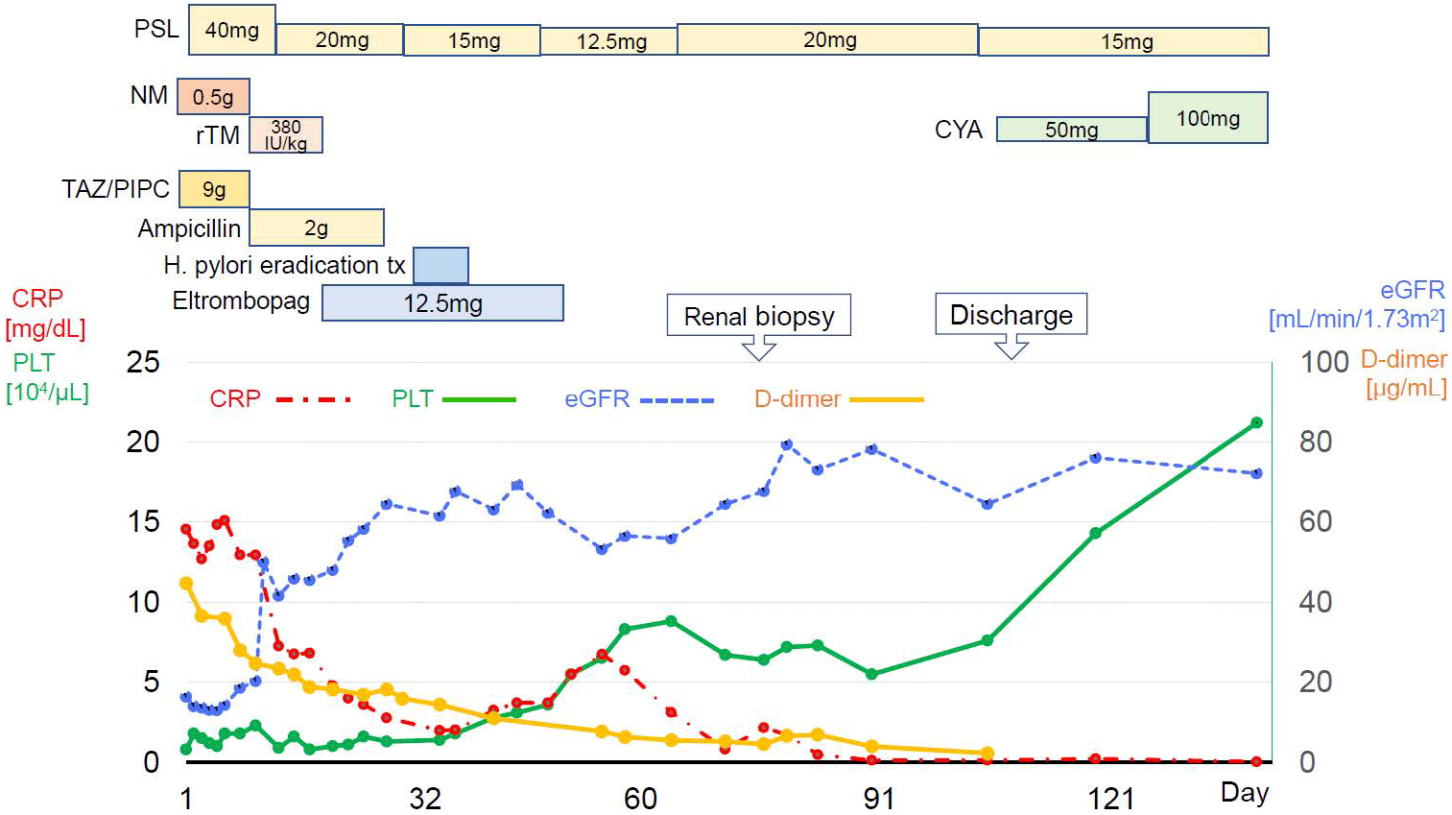
Clinical course since hospitalization. PSL, prednisolone; NM, Nafamostat mesylate; rTM, recombinant thrombomodulin; TAZ/PIPC, tazobactam/piperacillin; H.pylori, helicobacter pylori; CRP, c-reactive protein. PLT, platelet; eGFR, estimated glomerular filtration rate.

**Figure 3** summarizes the patient’s clinical course. The diagnosis of TAFRO syndrome was finally determined based on the clinical and laboratory findings presented here and the exclusion of other diseases that were essential for the diagnosis. After diagnosis of TAFRO syndrome, the patient discharged home on the 98^th^ hospital day. During the outpatient follow-up, cyclosporin (50mg/day) was initiated in addition to prednisolone. Subsequently, his platelet count gradually increased to nearly the expected value. Renal function was restored to normal.

## Discussion

Herein, we report a case of prolonged fever with thrombocytopenia that was resistant to antimicrobial treatment. This comprehensive investigation led to the diagnosis of TAFRO syndrome, which was difficult to differentiate from other diseases presenting with fever, prolonged thrombocytopenia, and severe organ damage during close examination and treatment of intestinal infections presenting with DIC.

The pathogenesis of TAFRO syndrome has not been fully elucidated; however its pathology can be explained by a cytokine storm, immune cell hyperactivation, and abnormalities in the immune system. As in the present case, the onset of TAFRO syndrome is often related to bacterial infection (6). Our patient had persistent diarrhea associated with an intestinal infection, which developed into enteral bacterial translocation and bacteremia. Bacterial infections trigger a cytokine storm, which leads to hypercoagulability and it is speculated that TAFRO syndrome develops subacutely with DIC (7, 8). There are rare but several reports of DIC complications in TAFRO syndrome (8–10). However, there have been no reports of cases such as the present case, in which DIC developed along with a bacterial infection and subsequently led to the diagnosis of TAFRO syndrome. The background leading to this complication has yet to be fully elucidated, but DIC has been proposed as a possible complication of TAFRO syndrome by involvement in the cytokine storm that is the pathogenesis of TAFRO syndrome (8, 11). Furthermore, cytokine-induced tissue factor activity and decreased thrombomodulin expression in vascular endothelial cells throughout the pathogenesis have been postulated, and subsequent thrombin production and thrombus formation may lead to DIC (7, 8, 11). The present case showed elevated levels of proinflammatory cytokines such as IL-6 and sIL-2R, as well as coagulation abnormalities. Although recombinant thrombomodulin is believed to inhibit thrombin formation as previously reported (8), its effects were limited in the present case.

It is challenging to differentially diagnose prolonged thrombocytopenia with a severe inflammatory response and reach a definitive diagnosis. A bone marrow biopsy enabled us to exclude other hematologic diseases that cause thrombocytopenia. Two bone marrow examinations did not detect reticulin myelofibrosis in this case, although this pathology is often recognized in patients with TAFRO syndrome. A report by Kurose et al. showed that reticulin myelofibrosis is not always evident in TAFRO syndrome, which also supports our diagnosis (12). Eltrombopag, a thrombopoietin receptor agonist that reacts with the receptor expressed on hematopoietic stem cells, in addition to corticosteroids, did not significantly restore the platelet count. Additionally, the patient tested positive for H. pylori antibodies, and eradication therapy mildly increased the platelet count but not as typically seen in cases of ITP. The histopathology of the bone marrow and its responsiveness to these treatments lowered the possibility of ITP. The reason for starting moderate steroids (0.5mg/kg/day) rather than high doses were used because we had not collected enough histopathologic specimens, and the steroids could interfere with the results and avoid causing excessive immunosuppression. Additionally, we withheld intravenous immune globulin (IVIG) because the patient tolerated steroids well and had no bleeding lesions that required immediate attention despite his severe thrombocytopenia.

The renal pathology results were consistent with TAFRO syndrome with a predominant endothelial cell lesion, which has been reported as a finding of renal TMA. Membranoproliferative glomerulonephritis (MPGN) and TMA-like findings without fibrin thrombi in the glomerular capillaries and arterioles are the two major histopathologies of TAFRO syndrome (13). Because MPGN and TMA shared similar histopathological findings such as mesangial proliferation, double contours of the glomerular basement membranes, and endocapillary proliferation on microscopy (14), differentiation between these should be performed on immunofluorescence and/or electron dense deposits by electron microscopy (15). The present case did not clinically show TMA findings, which are usually represented by schistocytes in blood smears and hemolytic anemia, and showed neither hypocomplementemia nor lowered ADAMTS-13 activity. Previous reports on renal TMA in TAFRO syndrome share a clinical presentation similar to that of the present case (16, 17).

Peripheral neuropathy, organomegaly, endocrinopathy, M protein levels, and skin changes (POEMS) syndrome is an important disorder that should be considered in the differential diagnosis of thrombocytopenia. However, our case showed no evidence of polyneuropathy or M-proteinemia, which are the major mandatory criteria for POEMS syndrome (18). The diagnostic level of serum VEGF for POEMS syndrome was more than 1,920 pg/mL (specificity 98%; sensitivity 73%) (19), indicating that the possibility of POEMS syndrome was low in this case.

As the effectiveness of combination therapy with cyclosporine and corticosteroids has been reported in previous cases of TAFRO syndrome (20), the platelet counts further increased after cyclosporine was added to the corticosteroids in this case. Cyclosporine, a widely used potent immunosuppressant, inhibits the activities of helper T cells and CD8+ T lymphocytes, and plays an important role in regulating multiple immune cell types and related inflammatory factors. It also regulates the proliferation and differentiation of T lymphocytes by inhibiting IL-2 and the expression of various proteins in dendritic cells, macrophages, and neutrophils. The IL-2-dependent pathway, in addition to IL-6, has recently been assumed to affect the etiology of TAFRO syndrome (20). Cyclosporine is also used to suppress the release of proinflammatory cytokines, including IL-2 (21). Its effect on the suppression of immune-medicated reactions was successful and yielded favorable results in this case.

In conclusion, we report a case of TAFRO syndrome that is complicated with DIC due to enterococcal bacteremia, and it was difficult to differentiate from other diseases, including SLE. There are several reports of TAFRO syndrome complicated with DIC. However, the present report is a rare case of TAFRO syndrome in DIC associated with infection. Although TAFRO syndrome is also a rare disease and its diagnosis is challenging, it can be encountered in daily clinical practice. TAFRO syndrome should always be considered a differential diagnosis for febrile and prolonged thrombocytopenia, and its possibility should be recalled, especially when the disease is complicated by many organ disorders and difficult to explain by a single condition. Alternatively, a definitive diagnosis for TAFRO syndrome should always be made with caution.

## Data availability statement

The original contributions presented in the study are included in the article. Further inquiries can be directed to the corresponding author.

## Ethics statement

Written informed consent was obtained from the individual(s) for the publication of any potentially identifiable images or data included in this article.

## Author contributions

HS: Conceptualization, Data curation, Writing – original draft. AK: Conceptualization, Data curation, Investigation, Methodology, Project administration, Validation, Writing – original draft, Writing – review & editing. MS: Data curation, Investigation, Writing – original draft. AE: Investigation, Methodology, Writing – original draft. HI: Investigation, Methodology, Writing – original draft. TO: Investigation, Methodology, Writing – original draft. YS: Conceptualization, Methodology, Writing – original draft. KS: Project administration, Writing – review & editing. TM: Supervision, Writing – review & editing. KF: Investigation, Project administration, Supervision, Writing – review & editing.
